# Probing the Migration of Free Radicals in Solid and Liquid Media via Cr(VI) Reduction by High-Energy Electron Beam Irradiation

**DOI:** 10.1038/s41598-018-33676-5

**Published:** 2018-10-12

**Authors:** Jie Han, Min Wang, Guilong Zhang, Furu Zhan, Dongqing Cai, Zhengyan Wu

**Affiliations:** 1 Key Laboratory of High Magnetic Field and Ion Beam Physical Biology, Chinese Academy of Sciences, Hefei, 230031 People’s Republic of China; 2 University of Science and Technology of China, Hefei, 230026 People’s Republic of China; 3 Key Laboratory of Environmental Toxicology and Pollution Control Technology of Anhui Province, Hefei Institutes of Physical Science, Chinese Academy of Sciences, Hefei, 230031 People’s Republic of China

## Abstract

To probe the migration of free radicals (FRs), the reduction behaviours of hexavalent chromium (Cr(VI)) in water and ice by high-energy electron beam (HEEB) irradiation were investigated. Interestingly, the reductive efficiency (RE) of Cr(VI) in water was appreciably higher than that in ice. Thus, it was proposed that the migration ability of FRs in water is distinctly higher than that in ice, likely because the migration performance of FRs is closely related to the intermolecular distance of water molecules. Furthermore, the RE of Cr(VI) in ice decreased gradually with the distance from the irradiated area, indicating that FRs could migrate in ice and that the migration performance was closely related to the RE. Additionally, FRs (hydrated electrons (${{\rm{e}}}_{{\rm{aq}}}^{-}$) and hydrogen radicals (·H)) generated during the irradiation process played a key role in the reduction of Cr(VI). Hydroxyl radicals (·OH) and H_2_O_2_ were the dominant negative factors for the reduction because of their oxidizing effects, but these factors could be eliminated by the removal of ·OH. This work reveals the migration performance of FRs in different media for the first time. This result may be useful for basic and applied studies in fields of environmental science related to FRs.

## Introduction

Free radicals (FRs), short-lived reactive intermediates in chemical reactions, play an important role in environmental pollutant treatment owing to their high redox capabilities^1–5^. FRs can be produced through many methods, including ultraviolet photolysis^6^, the Fenton reaction^7^, glow discharge plasma treatment^8^, and ionizing radiation^9^. Over the past few years, research on FRs has become one of the most popular topics in chemistry, focusing mainly on reaction dynamics^10^, chemical kinetics^11^, theoretical investigations^12^, and spectroscopy of FRs^13^, among other areas. However, little attention has been paid to the migration characteristics of FRs in different media because of the lack of convenient and suitable measurement methods for FRs, which possess a rather short half-life^14^. As a result, the migration behaviours of FRs are still unclear, creating an important gap limiting basic and applied studies of FRs in chemistry^15^. Therefore, it is important to develop a facile method for determining the migration of FRs.

FRs such as hydrated electrons (${{\rm{e}}}_{{\rm{aq}}}^{-}$), hydrogen radicals (·H) and hydroxyl radicals (·OH) can be produced from H_2_O radiolysis under high-energy electron beam (HEEB) irradiation^16,17^. In our previous study, we found that HEEB irradiation could significantly reduce hexavalent chromium (Cr(VI)), a typical heavy metal with a high risk of inducing cancer and gene mutation in human beings, to trivalent chromium (Cr(III)) with substantially lower toxicity^18,19^. Furthermore, it was deduced that ${{\rm{e}}}_{{\rm{aq}}}^{-}$, H, and OH generated during HEEB irradiation played important roles in the reduction process^19^. Importantly, the reductive efficiency (RE) of Cr(VI) was probably affected by the migration performance of FRs in different media. Consequently, the RE of Cr(VI) could be used as a key indicator for investigating the migration behaviour of FRs under HEEB irradiation.

In this work, the migration behaviours of FRs in water and ice were investigated under HEEB irradiation using the RE of Cr(VI) as an indicator. The results indicated that FRs migrated more actively in water than in ice and that the amount of FRs in ice decreased substantially with distance from the irradiated target. These findings have great value in promoting studies of FR-induced reactions in chemistry^15^, the bystander effect in radiation biology^20^, and other phenomena. Additionally, the effects of ${{\rm{e}}}_{{\rm{aq}}}^{-}$, ·H, and ·OH on the reduction of Cr(VI) under HEEB irradiation were investigated to determine the mechanism. Therefore, this work is also of great significance in promoting environmental studies on FRs.

## Results and Discussion

### Reduction performance investigation

**Figure 1:**
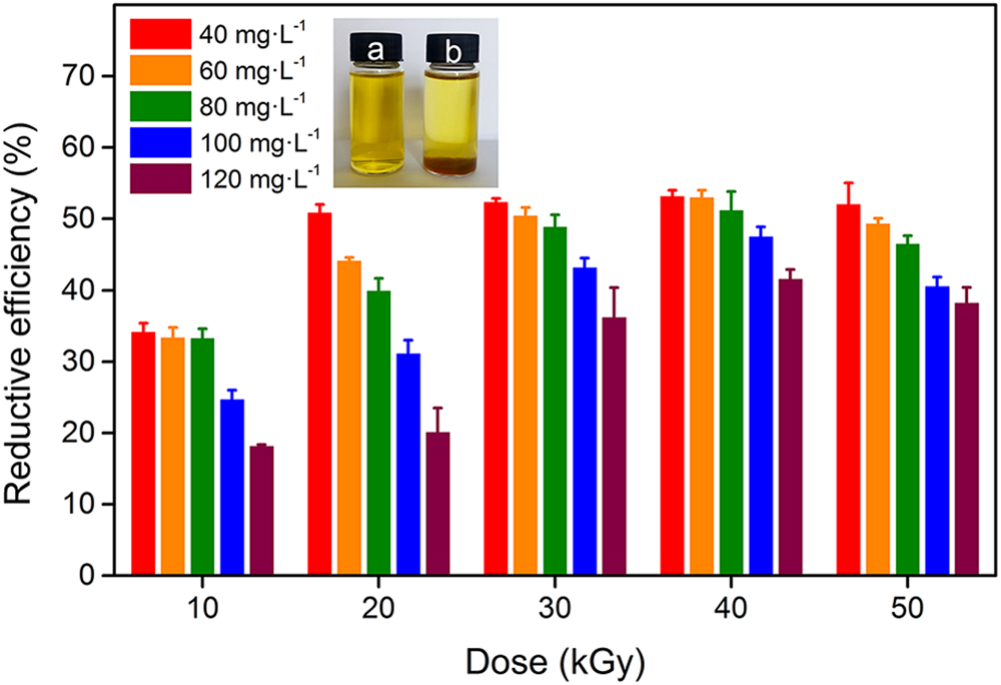
Reduction performance of HEEB irradiation on Cr(VI) in aqueous solution at pH of 4.5 with different initial concentrations. The insets (**a)** and (**b**) are digital images of the Cr(VI) aqueous solutions before and after irradiation, respectively. (Error bars indicate standard deviation (n = 3)).

The reduction of Cr(VI) in aqueous solution by HEEB irradiation was investigated. Figure 1 shows that with the increase of HEEB dose, the RE of Cr(VI) at different initial concentrations increased initially (<40 kGy), reaching the maximum value at 40 kGy, and then decreased (>40 kGy). In other words, 40 kGy was the optimum dose of HEEB for the reduction of Cr(VI) in aqueous solution. Generally, the RE of Cr(VI) decreased with the increase of the initial concentration of Cr(VI) at a certain HEEB dose. Notably, after irradiation, the yellow Cr(VI) aqueous solution became much lighter, and precipitates could be clearly observed at the bottom of the solution (insets in Fig. 1). The UV-Vis spectra of Cr(VI) aqueous solution before and after irradiation (40 kGy) were measured. As shown in Supplementary Fig. S1, after irradiation, the peak at 542 nm corresponding to the characteristic peak of Cr(VI) became weak, indicating that the concentration of Cr(VI) decreased after irradiation. The precipitation occurred because a large amount of Cr(VI) ions were reduced to insoluble Cr(III) by HEEB, which was proved by the following X-ray photoelectron spectrometer (XPS) and Fourier transform infrared (FTIR) analyses. The reduction performance of HEEB for Cr(VI) was attributed to reductive radicals such as ${{\rm{e}}}_{{\rm{aq}}}^{-}$ (e^−^ + nH_2_O = ${{\rm{e}}}_{{\rm{aq}}}^{-}$) and ·H (H_2_O = ·H + ·OH) generated from water radiolysis during the irradiation process. This result demonstrated that HEEB had a significant reduction capacity on Cr(VI) in aqueous solution and that this method is convenient and clean compared with traditional methods based on reducing agents such as nano zero-valent iron and amorphous FeS_2_^21,22^. Those methods have defects of complex procedure and secondary pollution.

**Figure 2:**
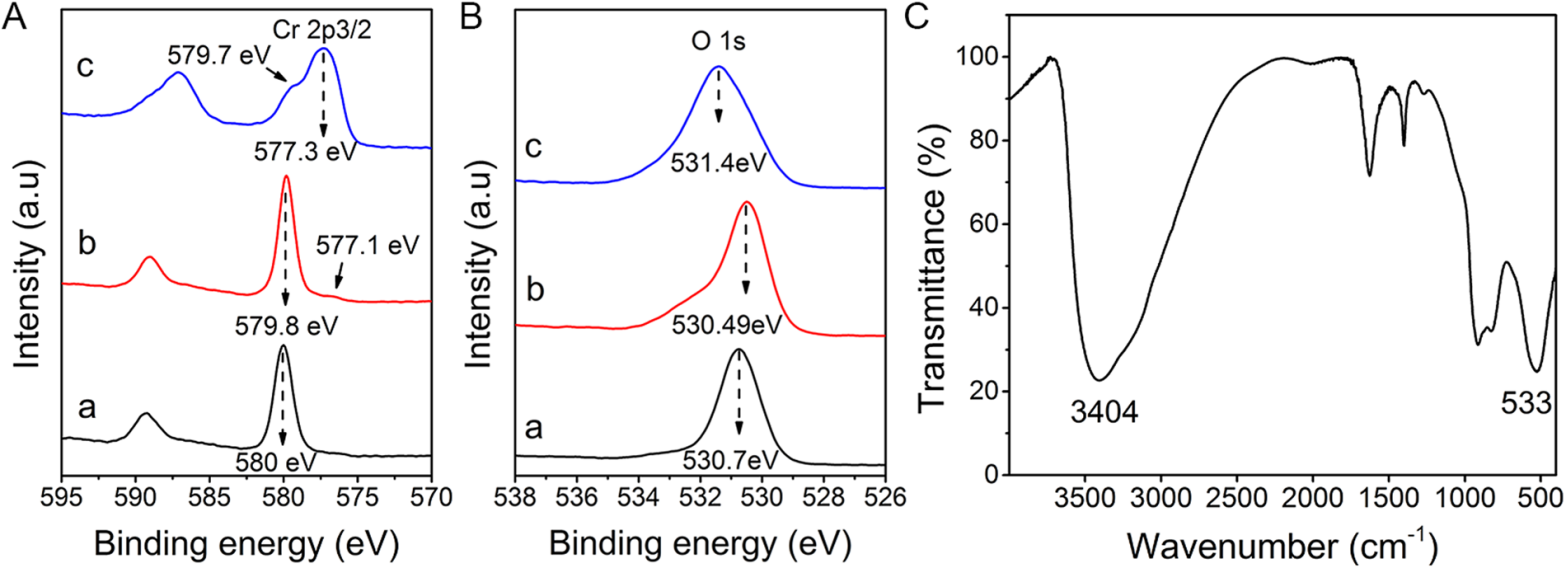
(**A**) Cr2p and (**B**) O1s XPS spectra of K_2_CrO_7_ (a), substances in supernatant (b), and precipitate (c) in HEEB-irradiated Cr(VI) aqueous solution. (**C**) FTIR spectrum of the precipitate (initial Cr(VI) concentration = 100 mg·L^−1^, dose = 40 kGy).

To determine the chemical states of Cr in solution after HEEB irradiation, the substances in the supernatant and precipitate were analysed by XPS. As shown in Fig. 2A, the strong peak at 579.8 eV and the weak peak at 577.1 eV in the spectrum of Fig. 2Ab correspond to the binding energies of 2p3/2 of Cr(VI) and Cr(III), respectively^23^, indicating that mainly Cr(VI) occurred in the supernatant together with a small amount of suspended Cr(III). In contrast, the XPS spectrum of Fig. 2Ac shows that the precipitate consisted mainly of Cr(III) and some Cr(VI), likely because some Cr(VI) ions were adsorbed onto the Cr(III) precipitate. As shown in Fig. 2B, the peak at 577.3 eV for Cr2p and the peak at 531.4 eV for O1s of the precipitate suggest that the main constituent of the precipitate was Cr(OH)_3_^24^. This conjecture was proved by FTIR analysis, as shown in Fig. 2C, wherein all peaks correspond to those of Cr(OH)_3_^25,26^. These results indicate that HEEB irradiation could effectively transform soluble CrO_7_^2−^ into insoluble Cr(OH)_3_, which could be easily removed from the solution.

### Effect of pH and temperature on Cr(VI) reduction

**Figure 3:**
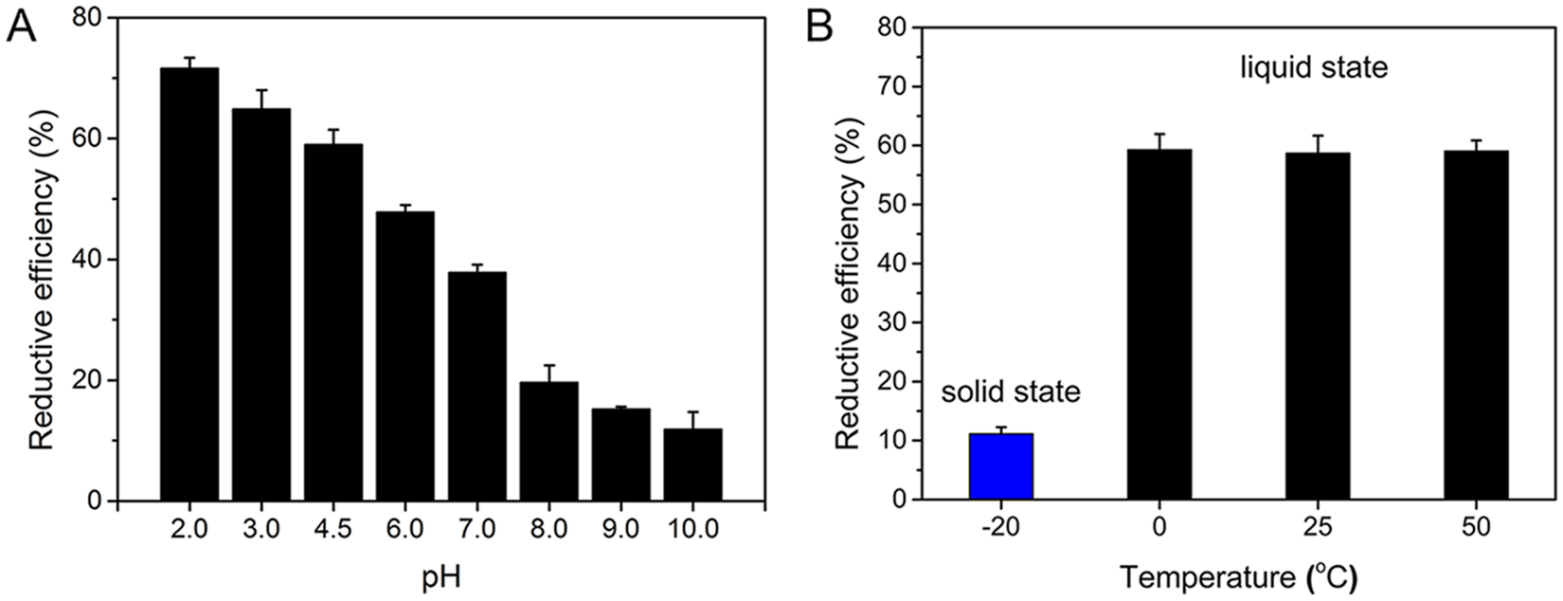
(**A**) Effect of pH on reduction performance of HEEB irradiation on Cr(VI) in aqueous solution at room temperature. (**B**) Reduction performance of HEEB irradiation on Cr(VI) at different temperatures at pH of 4.5 (initial Cr(VI) concentration = 100 mg·L^−1^, dose = 40 kGy). (Error bars indicate standard deviation (n = 3)).

The effect of pH on the reduction performance of HEEB irradiation for Cr(VI) was investigated. Figure 3A shows that the RE of Cr(VI) decreased substantially from 72% (pH = 2.0) to 12% (pH = 10.0) with increasing pH. On the one hand, this decrease occurred because the chemical state of Cr(VI) was greatly affected by pH, and HCrO_4_^−^ is the dominant form under acidic conditions, while CrO_4_^2−^ is the dominant form under neutral and alkaline conditions^27^. According to previous studies^28,29^, HCrO_4_^−^ possessed a higher oxidation capacity than CrO_4_^2−^, therefore HCrO_4_^−^ was more sensitive to reductive radicals than CrO_4_^2−^ was. On the other hand, pH can significantly affect the amount of ·OH generated in solution during the irradiation process^30^. According to the fact that ·OH can be formed from OH^−^ upon HEEB irradiation (equation (1)), more ·OH radicals were generated under alkaline conditions, resulting in a lower RE of Cr(VI). Therefore, this irradiation method is more suitable for the treatment of Cr(VI) under acidic condition.

1 $${{\rm{OH}}}^{-}\,\mathop{\longrightarrow }\limits^{{\rm{HEEB}}}\cdot \,{\rm{OH}}+{e}^{-}$$

In addition, the effect of temperature on the reduction performance of HEEB irradiation for Cr(VI) was investigated. As shown in Fig. 3B, there was no distinct difference in the RE of Cr(VI) in aqueous solution at 0, 25, and 50 °C, indicating that the activities of FRs were not appreciably affected by temperature between 0 and 50 °C. Interestingly, the RE (11%) of Cr(VI) in ice at −20 °C was much lower than that (approximately 58%) at temperatures of 0 °C or above. These results demonstrated that the activities of FRs toward the reduction of Cr(VI) in water were significantly higher than those in ice, likely because of the higher migration performance of FRs in water compared with that in ice.

### Probing the migration performance of FRs in different media

**Figure 4:**
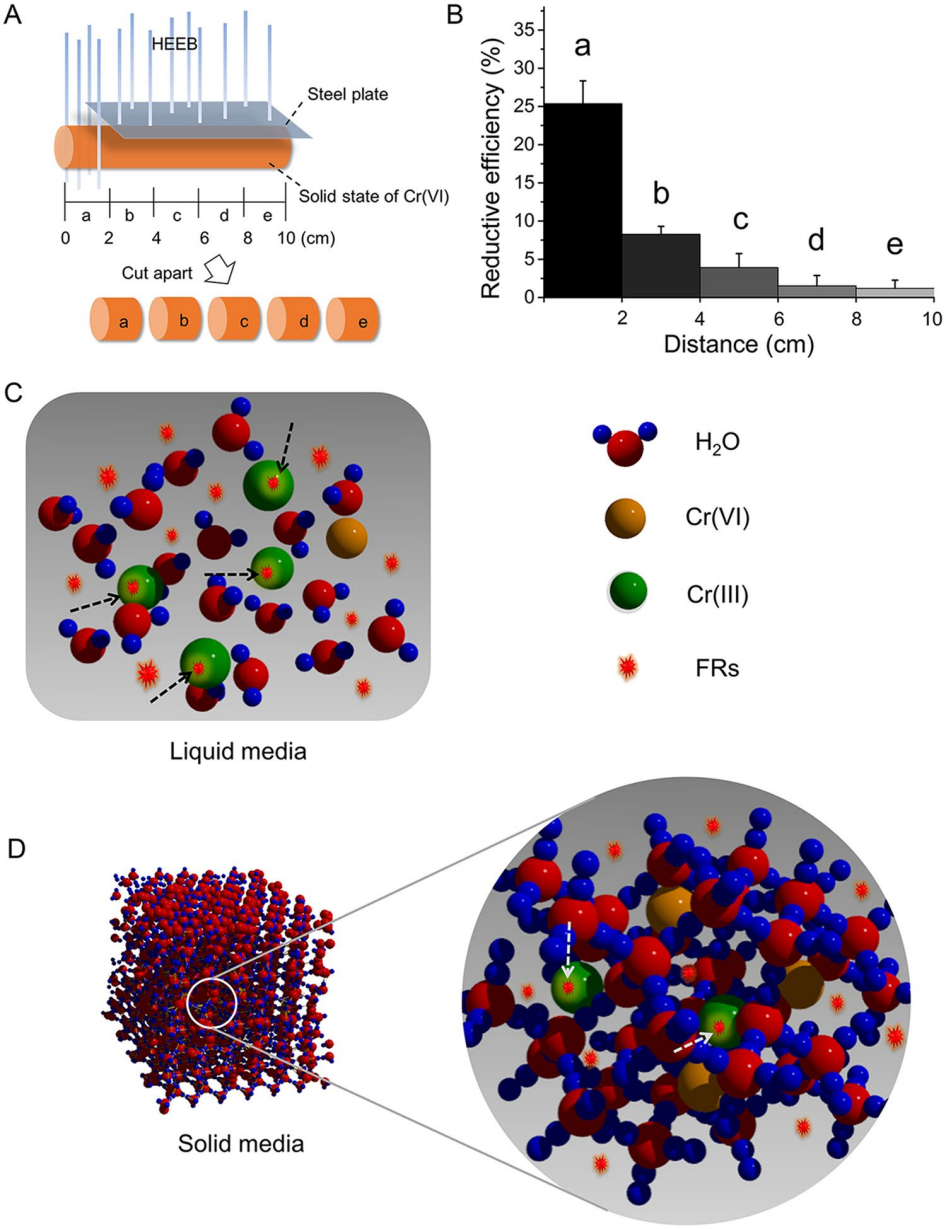
(**A**) Schematic diagram of the experimental system of HEEB irradiation on ice-Cr(VI). (**B**) Reduction performance of Cr(VI) in different parts of HEEB-irradiated ice-Cr(VI) (Initial Cr(VI) concentration = 100 mg·L^−1^, dose = 40 kGy) (Error bars indicate standard deviation (n = 3)). (**C**,**D**) Schematic diagrams of FRs in water and ice, respectively.

To determine the relationship between the reduction activity and migration behaviour of FRs, the reduction performance of Cr(VI) in a partially irradiated ice cylinder was investigated (Fig. 4A). As shown in Fig. 4B, the RE of Cr(VI) in ice decreased with distance from the irradiated region. Notably, the RE of Cr(VI) in the irradiated part (Fig. 4Aa) was significantly higher than that in the nonirradiated part nearby (Fig. 4Ab). During the irradiation process, FRs were generated in the irradiated part of the ice cylinder, some of which could migrate to other parts, and the amount of FRs likely decreased with distance from the irradiated part. The decrease in the amount of FRs with distance resulted in a decrease in the contact probability between FRs and Cr(VI), thus decreasing the RE of Cr(VI). Therefore, the migration performance of FRs played a key role in the reductive activity on Cr(VI).

To interpret the large difference in Cr(VI) RE observed between water and ice, two molecular models of the states of FRs in water and ice were proposed, as shown in Fig. 4C,D. In water, the H_2_O molecules aligned loosely and irregularly^31^ such that the intermolecular distance was large enough for the migration of FRs, resulting in high migration performance of FRs among water molecules, a high contact probability between FRs and Cr(VI) and thus a high RE for Cr(VI). In contrast, the H_2_O molecules in ice aligned regularly to form a crystal structure^31,32^, wherein the intermolecular distance was too small for the migration of FRs among the molecules. This arrangement was unfavourable for the contact between FRs and Cr(VI), resulting in a lower RE for Cr(VI). In other words, FRs displayed a higher migration capacity in water than in ice, and the migration performance of FRs was mainly determined by the H_2_O molecule alignment and intermolecular distance.

### Effect of FRs on Cr(VI) reduction

**Figure 5:**
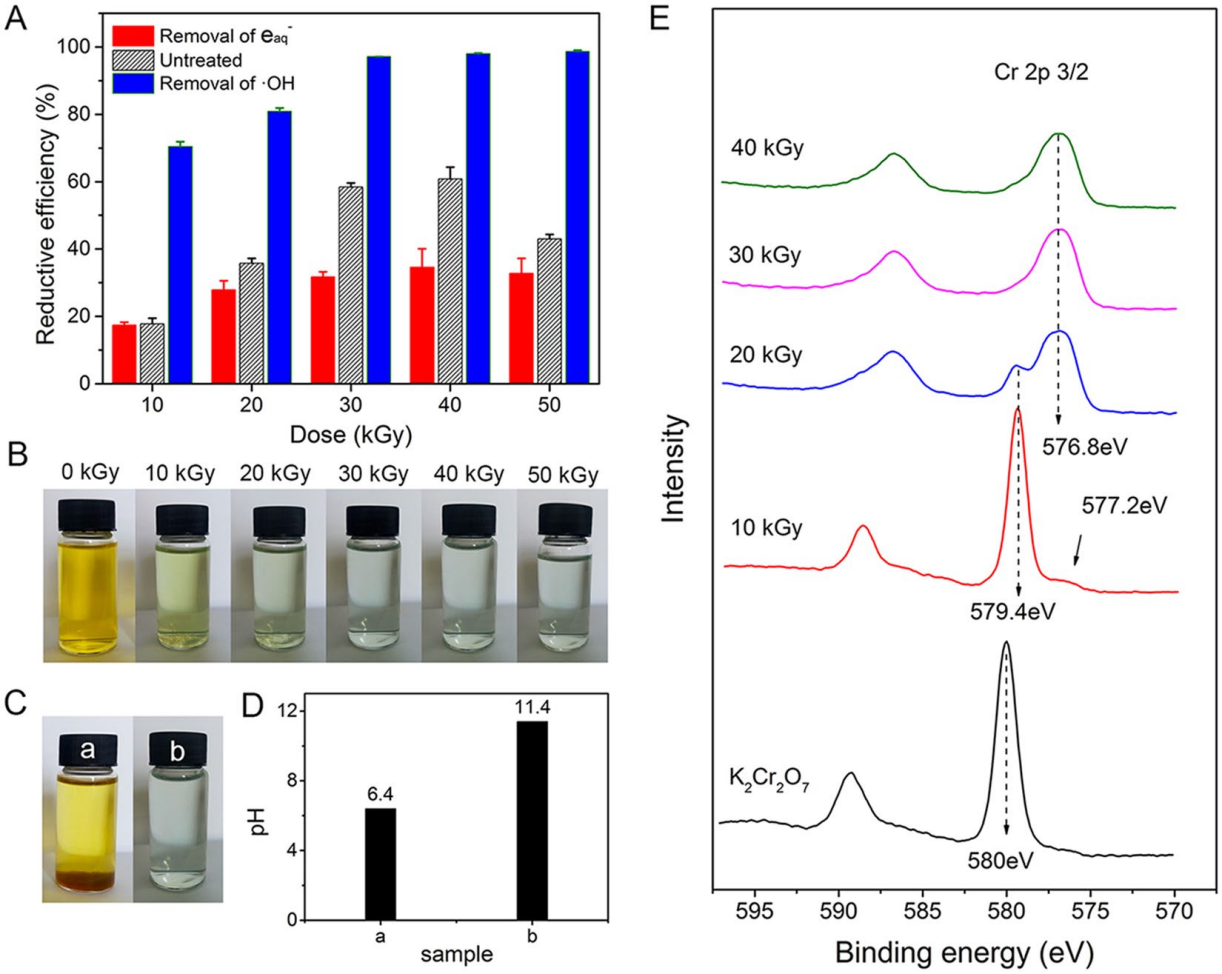
(**A**) Effect of ${{\rm{e}}}_{{\rm{aq}}}^{-}$ and OH on RE of Cr(VI) in aqueous solution (Error bars indicate standard deviation (n = 3)). (**B**) Digital photographs of irradiated Cr solutions in the absence of ·OH. (**C**) Digital photographs of irradiated Cr solutions (a) and irradiated Cr solutions in the absence of ·OH (b) with a dose of 40 kGy. (**D**) pH of irradiated Cr solutions (a) and irradiated Cr solutions in the absence of ·OH (b) with a dose of 40 kGy. (**E**) XPS spectra of the substances in the supernatants of these irradiated solutions in the absence of OH (initial Cr(VI) concentration = 100 mg·L^−1^).

The effect of ${{\rm{e}}}_{{\rm{aq}}}^{-}$ and ·OH on the RE of HEEB irradiation on Cr(VI) in aqueous solution was investigated. Figure 5A shows that the RE of Cr(VI) by HEEB at all doses decreased after the removal of ${{\rm{e}}}_{{\rm{aq}}}^{-}$, while it clearly increased after the removal of ·OH compared with that without the removal of FRs. Notably, although ${{\rm{e}}}_{{\rm{aq}}}^{-}$ in the solution were removed, a large number of Cr(VI) ions were still reduced by HEEB at all doses. These results indicate that both ${{\rm{e}}}_{{\rm{aq}}}^{-}$ and ·H played key roles in the reduction of Cr(VI) according to equations (2) and (3). Additionally, ·OH was the dominant negative factor for the reduction of Cr(VI), because of its ability to oxidize ${{\rm{e}}}_{{\rm{aq}}}^{-}$, ·H, and Cr(III), as shown in equations (4–6).

2 $$3{{{\rm{e}}}_{{\rm{aq}}}}^{-}+{\rm{Cr}}({\rm{VI}})\to {\rm{Cr}}({\rm{III}})$$

3 $$3\,\cdot \,{\rm{H}}+{\rm{Cr}}({\rm{VI}})\to {\rm{Cr}}({\rm{III}})+3{{\rm{H}}}^{+}$$

4 $$3\,\cdot \,{\rm{OH}}+{\rm{Cr}}({\rm{III}})\to {\rm{Cr}}({\rm{VI}})+3{{\rm{OH}}}^{-}$$

5 $$\cdot {\rm{OH}}+{{{\rm{e}}}_{{\rm{aq}}}}^{-}\to {{\rm{OH}}}^{-}$$

6 $$\cdot {\rm{OH}}+\cdot \,{\rm{H}}\to {{\rm{H}}}_{2}{\rm{O}}$$

After the removal of ·OH, HEEB irradiation could transform the yellow Cr(VI) solution into a green solution with green Cr(III) precipitates (Fig. 5B). Interestingly, the amount of Cr(III) precipitate after the removal of ·OH was significantly lower than that with ·OH because of the different pH levels of the resulting solutions: the pH of the former was 11.4, while that of the latter was 6.4 (Fig. 5C,D). Clearly, pH has a strong effect on the state of Cr, wherein Cr(III) exists as insoluble Cr(OH)_3_ at pH levels of 6.4–10, while it exists as soluble Cr(OH)_4_^−^ at pH levels of 10–14^33^. Therefore, the amount of Cr(III) precipitate after the removal of ·OH was less than that with ·OH. To determine the form in which Cr existed in the irradiated solution in the absence of ·OH, XPS analysis of the substances in the supernatant were performed as shown in Fig. 5E. The peaks at 576.8 and 577.2 eV correspond to the binding energy of 2p3/2 of Cr(III), and the peak at 579.4 eV corresponds to the binding energy of 2p3/2 of Cr(VI). The spectra show that Cr mainly existed in the form of Cr(VI) in the supernatant of the irradiated Cr(VI) solutions at 10 kGy. With the increase of the dose, the peak at 579.4 eV weakened, while the peaks at 576.8 and 577.2 eV became much stronger, suggesting that the amount of Cr(VI) in the supernatant decreased while the amount of Cr(III) gradually increased. When the dose was greater than 30 kGy, all Cr(VI) in the supernatant was reduced to Cr(III), consistent with the results shown in Fig. 5A.

### Stability investigation of Cr(III)

**Figure 6:**
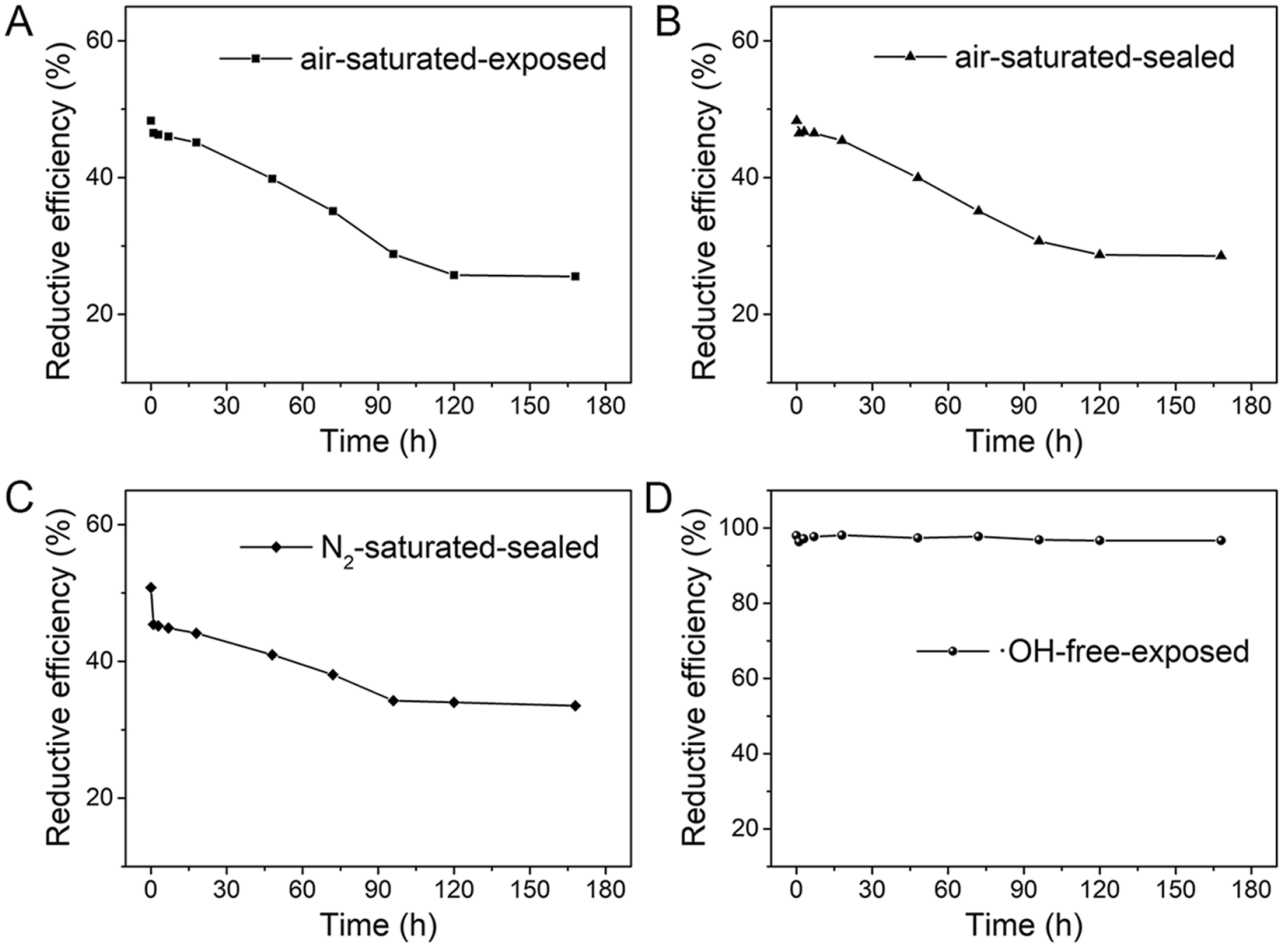
(**A**,**B**) RE of Cr(VI) over time in air-saturated solution kept exposed to air and sealed after irradiation. (**C**) RE of Cr(VI) over time in N_2_-saturated solution kept sealed after irradiation. (**D**) RE of Cr(VI) over time in·OH-free solution kept exposed to air after irradiation (initial Cr(VI) concentration = 100 mg·L^−1^, dose = 40 kGy).

After irradiation, the stabilities of Cr(III) were investigated in an air-saturated solution exposed to air (air-saturated-exposed), a sealed air-saturated solution (air-saturated-sealed), a sealed, N_2_-saturated solution (N_2_-saturated-sealed), and a ·OH-free solution exposed to air (·OH-free-exposed). Figure 6A–C clearly shows that the REs of Cr(VI) in the air-saturated-exposed, air-saturated-sealed, and N_2_-saturated-sealed solutions displayed similar decreasing trends, indicating that some of the Cr(III) could be oxidized to Cr(VI) and that this oxidization process was weakly related to the air inside and outside the solutions. In other words, air was not the dominant reason for the oxidation of Cr(III). However, Cr(VI) remained rather stable over time in the ·OH-free-exposed solution, suggesting that Cr(III) could not be oxidized to Cr(VI) in the absence of ·OH. That is, ·OH or the secondary particles were likely the dominant factors affecting the oxidation of Cr(III) to make it unstable.

**Figure 7:**
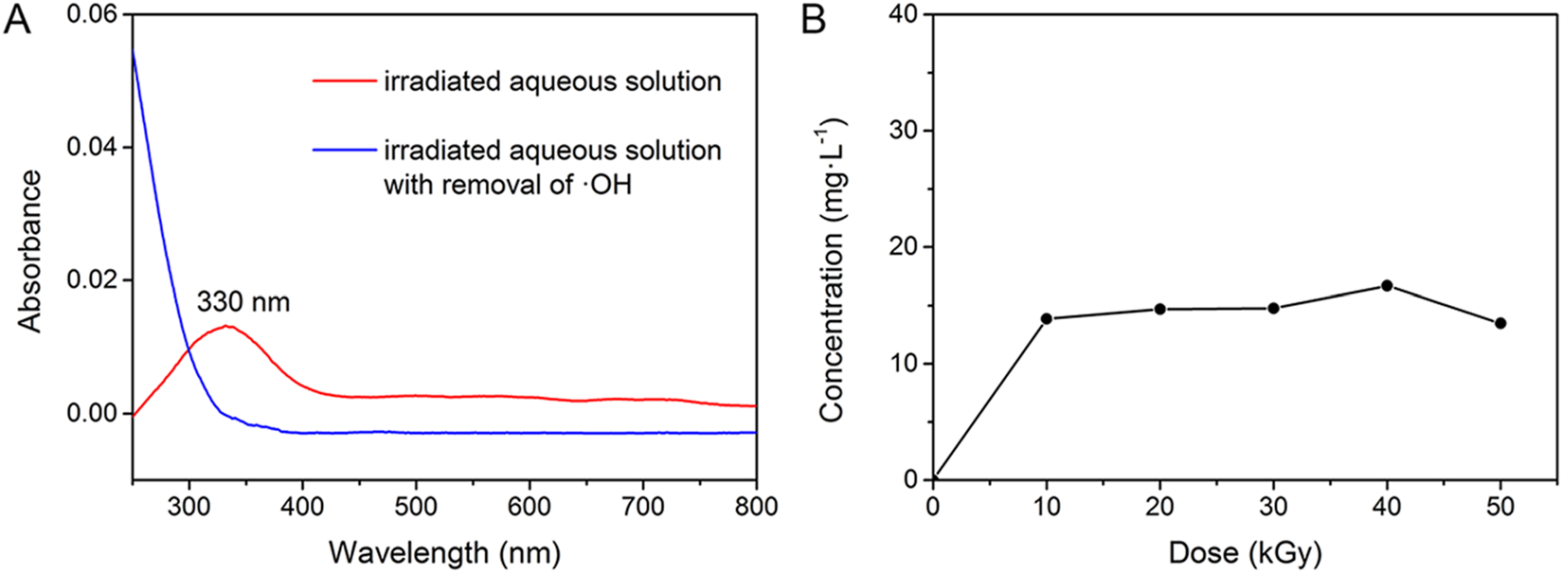
(**A**) UV-Vis absorption spectra of H_2_O_2_ in irradiated H_2_O with a dose of 40 kGy. (**B**) Effect of HEEB irradiation on H_2_O_2_ concentration in aqueous solution.

In fact, the short-lived ·OH generated during the irradiation process can react with each other to form H_2_O_2_, as indicated by equation (7)^30,34^ and as proved by the UV-Vis absorption spectra in Fig. 7A. The characteristic absorption peak (330 nm) for H_2_O_2_ appeared in the absorption spectrum of the irradiated aqueous solution, indicating that H_2_O_2_ was generated and could last for a long time after irradiation. Moreover, with the increase of HEEB dose, the H_2_O_2_ concentration in the aqueous solution increased rapidly initially (<10 kGy), increased slowly (10–40 kGy), and then decreased (>40 kGy). The maximum was approximately 17 mg·L^−1^. The long-lived H_2_O_2_ was likely the dominant negative factor affecting the stability of Cr(III); however, this factor could be eliminated after the removal of ·OH.

7 $$\cdot {\rm{OH}}+\cdot \,{\rm{OH}}\to {{\rm{H}}}_{2}{{\rm{O}}}_{2}$$

### Mechanism study

**Figure 8:**
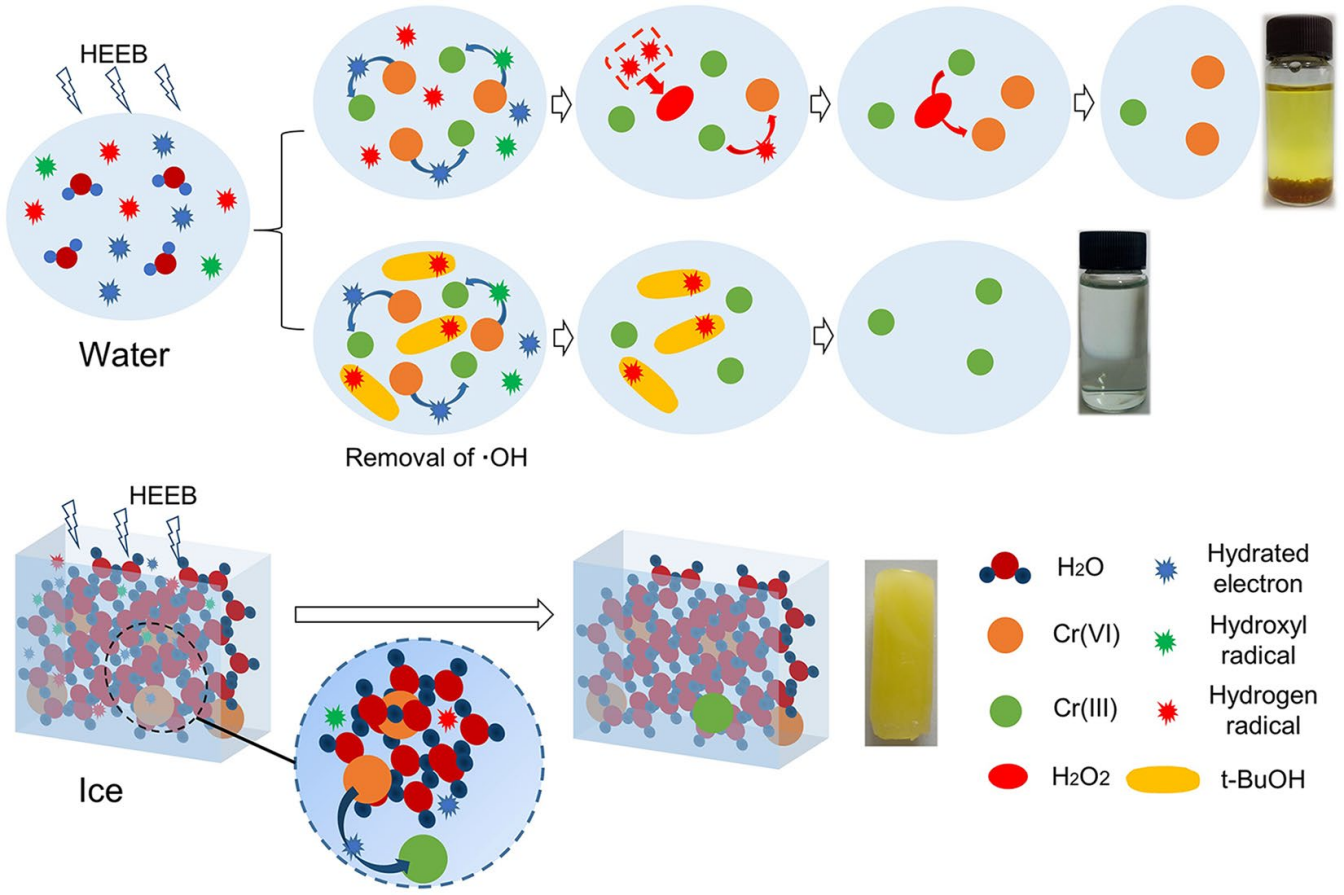
Schematic diagram of the reduction mechanism of HEEB irradiation on Cr(VI) in water and ice.

Based on the preceding analyses, the reduction of FRs on Cr(VI) occurred as indicated by the schematic diagram shown in Fig. 8. FRs (${{\rm{e}}}_{{\rm{aq}}}^{-}$, ·H, and ·OH) were generated instantaneously in Cr(VI) aqueous solution from H_2_O radiolysis during the HEEB irradiation process. Due to the larger intermolecular distance of water compared with that of ice, FRs could migrate freely in water; thus, the reductive FRs (${{\rm{e}}}_{{\rm{aq}}}^{-}$ and ·H) could efficiently reduce Cr(VI) to Cr(III), resulting in a higher RE in water compared with that in ice. Consequently, part of the obtained Cr(III) could be oxidized to Cr(VI) by ·OH and H_2_O_2_ to make Cr(III) unstable; however, this problem could be solved through the removal of ·OH by adding t-BuOH before HEEB irradiation.

## Conclusions

The reduction performance of HEEB irradiation for Cr(VI) in water and ice was investigated. The results indicated that HEEB could effectively reduce Cr(VI) to Cr(III) and that FRs (${{\rm{e}}}_{{\rm{aq}}}^{-}$ and ·H) generated during the irradiation process were the dominant reason for the reduction. It was found that the migration of FRs was likely determined by the molecular alignment of the media, and the migration performance of FRs greatly affected the reductive activity on Cr(VI). Owing to the larger molecular distance of water compared with that of ice, ${{\rm{e}}}_{{\rm{aq}}}^{-}$ and ·H could migrate more easily in water and thus displayed better reductive capacity on Cr(VI) than in ice. Additionally, the obtained Cr(III) was unstable because of the oxidization effect of ·OH and H_2_O_2_; however, this problem could be solved after the removal of ·OH. This work reveals different migration behaviours of FRs in the liquid and solid phases, which can promote basic and applied studies related to FRs.

## Methods

### Materials

K_2_Cr_2_O_7_ and other chemicals were of analytical grade and purchased from Sinopharm Chemical Reagent Company (Shanghai, China). Deionized water was used throughout this work.

### RE of HEEB irradiation for Cr(VI) in aqueous solution

Cr(VI) aqueous solutions with concentrations of 40, 60, 80, 100, and 120 mg·L^−1^ were placed in sealed centrifuge tubes, which were then irradiated by a HEEB accelerator (10 MeV and 10 kW) (IHI10, IHI Co., Japan) with doses of 10, 20, 30, 40, and 50 kGy at different temperatures (−20, 0, 25, and 50 °C) under varying pH conditions (2.0 to 10.0). The concentration of the Cr(VI) remaining in solution was then determined by the diphenylcarbazine (DPC) method^25^. All experiments were performed in triplicate. The RE of Cr(VI) was calculated according to equation (8):

8 $${\rm{RE}}\,( \% )=({{\rm{C}}}_{0}-{{\rm{C}}}_{{\rm{t}}})/{{\rm{C}}}_{{\rm{0}}}\times 100 \% $$

where C0 and Ct are the initial and resulting Cr(VI) concentrations (mg·L^−1^), respectively.

### RE of HEEB irradiation for Cr(VI) in ice

Cr(VI) aqueous solution (100 mg·L^−1^) was frozen into an ice-Cr(VI) cylinder (length of 10 cm and diameter of 1 cm) in a PVC tube at −20 °C, and one end (8 cm) of the resulting tube was covered by a steel plate with a thickness of 2 cm. The resulting system was then laid flat and irradiated by HEEB with a dose of 40 kGy such that only the uncovered region (2 cm) of the tube could be effectively irradiated. Subsequently, the ice-Cr(VI) was evenly cut into five parts, with each measuring 2 cm long, and the concentrations of Cr(VI) in these parts were measured after melting to obtain the respective REs.

### Effect of FRs on Cr(VI) reduction

To investigate the effect of FRs on the reduction performance of HEEB, ${{\rm{e}}}_{{\rm{aq}}}^{-}$ and ·OH, the dominant radicals in irradiated aqueous solution, were selectively scavenged during HEEB irradiation. For ${{\rm{e}}}_{{\rm{aq}}}^{-}$, 50 mL Cr(VI) aqueous solution (pH = 4.5, 100 mg·L^−1^) was placed in an uncovered stainless-steel cup, which was placed on a grounded iron conveyor, and then treated by HEEB irradiation so that the ${{\rm{e}}}_{{\rm{aq}}}^{-}$ generated during the irradiation process could discharge immediately through the cup and conveyor. To remove ·OH generated during irradiation, 3 mL of tert-butanol (t-BuOH) (98%) was added to 50 mL of N_2_-saturated Cr(VI) aqueous solution (pH = 4.5, 100 mg·L^−1^) before irradiation according to equation (9)^35^. After irradiation, the concentrations of Cr(VI) were determined to obtain the REs.

9 $${({{\rm{CH}}}_{3})}_{3}{\rm{COH}}+\cdot \,{\rm{OH}}=\cdot \,{{\rm{CH}}}_{2}{({{\rm{CH}}}_{3})}_{2}{\rm{COH}}+{{\rm{H}}}_{2}{\rm{O}}$$

### Stability investigation of Cr(III) after HEEB treatment

Cr(VI) aqueous solutions (100 mg·L^−1^) were placed in centrifuge tubes, and the solutions were saturated with N_2_ or air. The tubes were then sealed and irradiated by HEEB with a dose of 40 kGy. Afterwards, the N_2_-saturated tubes were kept sealed. One of the air-saturated tube was kept sealed and the other was exposed to air; the Cr(VI) concentrations were determined at a given time to determine the stability of Cr(III) in the HEEB-treated solution. Additionally, to determine the effect of ·OH on the stability of Cr(III), 3 mL of t-BuOH (98%) was added to 50 mL of N_2_-saturated Cr(VI) aqueous solution (pH = 4.5, 100 mg·L^−1^) before irradiation to remove ·OH generated during irradiation. After irradiation (40 kGy), the solution was exposed to air, and the Cr(VI) concentration was determined at a given time.

### Characterization

Structure and composition analyses were conducted using a FTIR spectrometer (iS10, Nicolet Co., USA) and an XPS (ESCALAB 250, Thermo-VG Scientific Co., USA). The concentrations of Cr(VI) and H_2_O_2_ were measured using a UV-Vis spectrophotometer (UV 2550, Shimadzu Co., Japan) at wavelengths of 540 and 330 nm^25,36^.

## Electronic supplementary material

Supplementary Information
