# Supplementary material for: Probing the Migration of Free Radicals in Solid and Liquid Media via Cr(VI) Reduction by High-Energy Electron Beam Irradiation
Source: Sci Rep. 2018 Oct 12;8:15196. doi: 10.1038/s41598-018-33676-5 (PMC6185987; doi:10.1038/s41598-018-33676-5)
Supplement: Supplementary file 1 — Supplementary Information [file 41598_2018_33676_MOESM1_ESM.pdf]

Supplementary Information

Probing the Migration of Free Radicals in Solid and Liquid Media via Cr(VI) Reduction by High-Energy Electron Beam Irradiation

Jie Han[1,2], Min Wang[1,2], Guilong Zhang[1,3], Furu Zhan[1,3,]*, Dongqing Cai[1,3,]*, and Zhengyan Wu[1,3,]*

[1]Key Laboratory of High Magnetic Field and Ion Beam Physical Biology, Chinese Academy of Sciences, Hefei 230031, People's Republic of China

[2]University of Science and Technology of China, Hefei 230026, People's Republic of China

[3]Key Laboratory of Environmental Toxicology and Pollution Control Technology of Anhui Province, Hefei Institutes of Physical Science, Chinese Academy of Sciences, Hefei 230031, People's Republic of China

*Corresponding Authors

F.Z. Tel.: +86-551-65595012, Fax: +86-551-65595012. E-mail: zfr@ipp.ac.cn.

D.C. Tel.: +86-551-65595012, Fax: +86-551-65595012. E-mail: dqcai@ipp.ac.cn.

Z.W. Tel.: +86-551-65595012, Fax: +86-551-65595012. E-mail: zywu@ipp.ac.cn.

Number of figure: 1

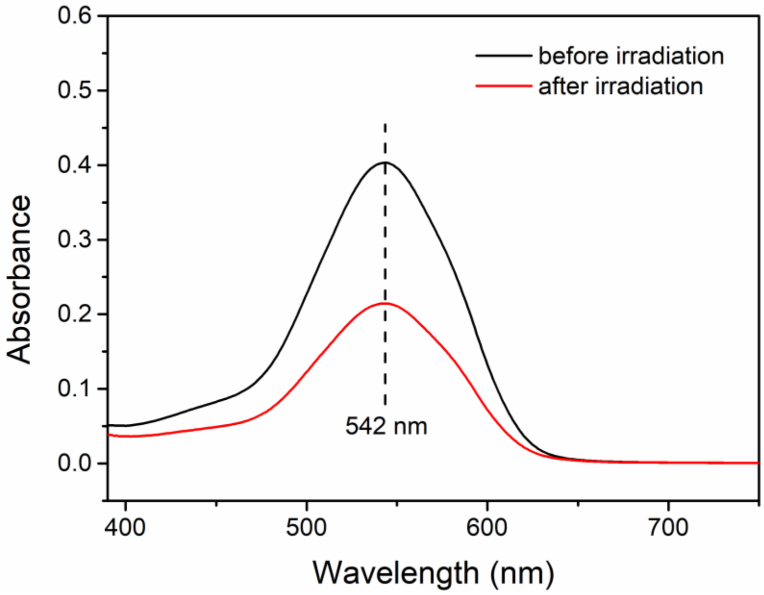

Figure S1. UV-Vis spectra of Cr(VI) aqueous solution before and after irradiation (Initial Cr(VI) concentration=100 mg·L^{-1}, dose=40 kGy).
